# Mechanism of a polyherbal mixture alleviates calf diarrhea: an integrated network pharmacology, metabolomics, and microbiome study

**DOI:** 10.1186/s40104-026-01425-8

**Published:** 2026-05-31

**Authors:** Shaoxiong Ji, Jianmin Xia, Zhantao Yang, Siyuan Liu, Xinyue Zhang, Xiaojing Liu, Yangyi Hao, Wei Wang, Shenfei Long, Shengli Li

**Affiliations:** 1 State Key Laboratory of Animal Nutrition and Feeding, College of Animal Science and Technology, China Agricultural University, Beijing, 100193 China; 2 Beijing Jingwa Agricultural Science & Technology Innovation Center, Beijing, 101206 China

**Keywords:** Diarrhea, Molecular docking, Network pharmacology, Polyherbal mixtures

## Abstract

**Background:**

Calf diarrhea represents a prevalent and serious challenge in dairy farming. While medicinal plants demonstrate considerable potential for preventing calf diarrhea within antibiotic-free farming systems, their active components and mechanisms remain poorly characterized. The objective of this study was to investigate a polyherbal mixture (PM; including *Crataegus pinnatifida*, *Callicarpa nudiflora* Hook. & Arn., *Mallotus apelta *(Lour.) Müll.Arg*.*, *Amomum villosum* Lour., *Centella asiatica* (L.) Urban, and *Alpinia oxyphylla* Miq.) supplemented to preweaning calves from d 4 to 60, utilizing an integrated approach combining network pharmacology, metabolomics, and microbiomics.

**Results:**

Dietary supplementation with 40 g/d of PM significantly decreased the occurrence of diarrhea (*P* < 0.05), increased monocyte levels (*P* < 0.05), and improved jejunal villus height (*P* < 0.05). Network pharmacology predicts that IL6, EGFR, SRC, TP53, and CCND1 are key targets, while acacetin, chrysin, tectochrysin, dihydroartemisinic acid, and lysionotin may be potential active constituents. The serum metabolome revealed that PM supplementation significantly enriched the steroid hormone biosynthesis. At the same time, PM altered the gastrointestinal microbiota, increasing the abundance of bacteria such as *Mediterranea massiliensis*, *Prevotella denticola*, and *Duncaniella freteri* in the rumen and *Clostridium nexile* in feces, while decreasing the abundance of *Blautia producta*, *Vescimonas fastidiosa*, and *Alistipes putredinis* in feces (*P* < 0.05).

**Conclusions:**

Collectively, these findings suggest that PM supplementation alleviated calf diarrhea by remodeling serum steroid hormone biosynthesis and improving ruminal and fecal microbiota composition. Acacetin, chrysin, tectochrysin, dihydroartemisinic acid, and lysionotin may be potential active components.

**Graphical Abstract:**

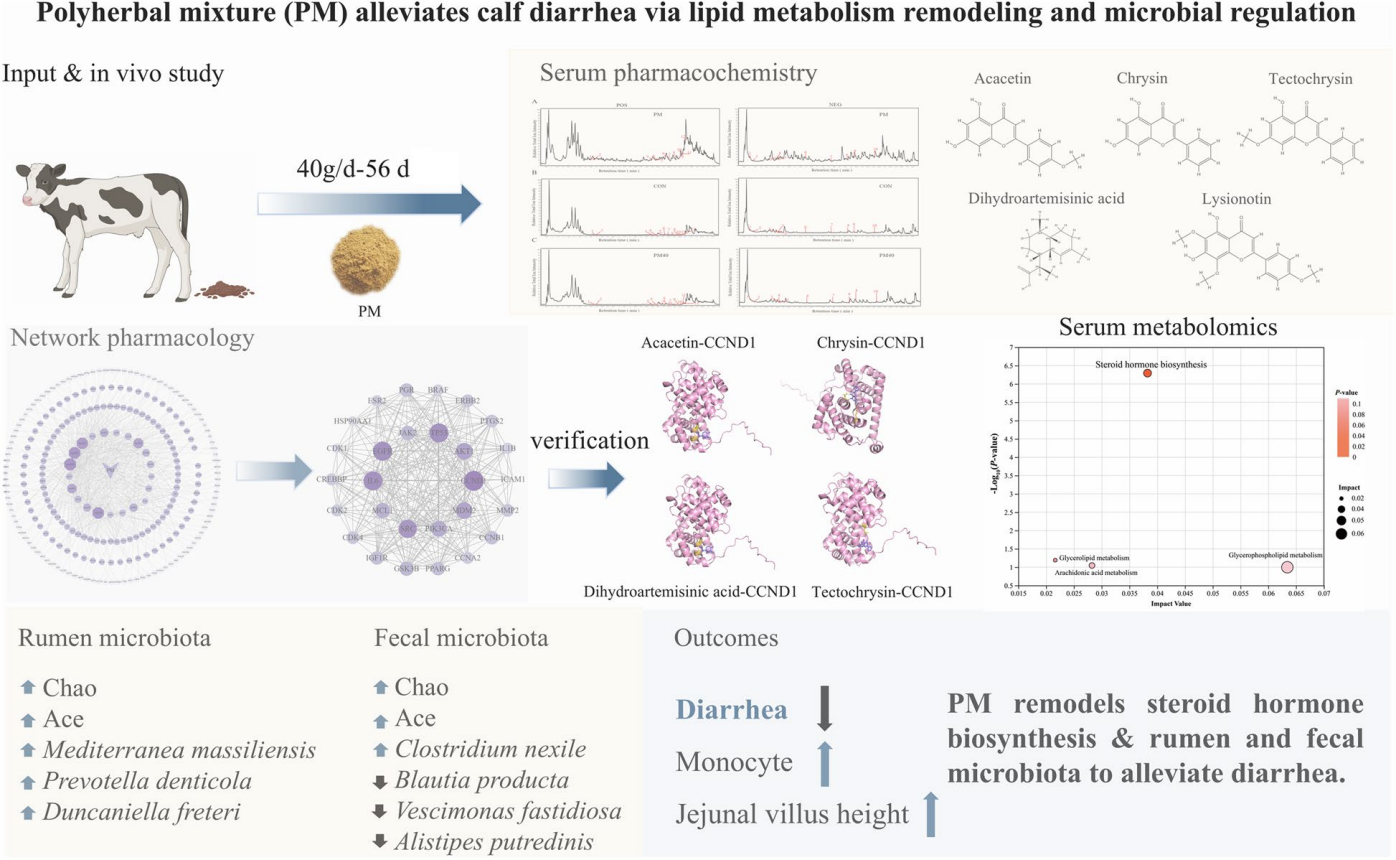

**Supplementary Information:**

The online version contains supplementary material available at 10.1186/s40104-026-01425-8.

## Introduction

Calf diarrhea is widely recognized as one of the most prevalent and serious diseases affecting dairy farming operations globally [1], causing high mortality rates [2], growth retardation [3], and long-term impairment of production performance [3], leading to significant economic losses in the industry [4, 5]. Although antibiotic therapy remains the primary clinical strategy for managing calf diarrhea [6, 7], prolonged or indiscriminate use contributes to the emergence of antimicrobial resistance and disrupts the gut microbiota, potentially exacerbating health and environmental challenges [8, 9]. Therefore, developing safe, effective, and sustainable antibiotic alternatives has become an urgent priority for the livestock industry.

Research into medicinal plants reveals their potential as viable antibiotic alternatives for managing calf diarrhea [10, 11]. Empirical evidence, including studies by Jahani-Azizabadi and Turini, demonstrates that specific herbal blends and traditional formulations can effectively reduce the incidence of diarrhea and shorten its duration [12, 13]. Similarly, Ding et al. [14] demonstrate that a traditional Chinese medicine formulation, when synergized with a bacteriocin, exhibited potent antibacterial activity against key pathogens like *Escherichia coli* and *Salmonella*, while also alleviating inflammation and modulating gut microbiota in vivo. Despite these encouraging phenotypic outcomes, the pharmacological characterization of herbal preparations remains challenging. Their complex multi-component nature and polypharmacological profiles complicate the comprehensive elucidation of their active constituents and mechanisms of action using conventional single-target experimental models [15]. Most existing studies have primarily focused on phenotypic outcomes, whereas systematic investigations into their effects on active constituents and signaling pathways remain limited.

The pathogenesis of preweaning calf diarrhea involves multiple interconnected factors, including systemic metabolic dysregulation and gut microbiota imbalance [16, 17]. Compared with single-ingredient interventions, multi-herb formulations may offer synergistic benefits by simultaneously modulating multiple physiological pathways [18]. Based on the principles of Traditional Chinese Veterinary Medicine (TCVM), which emphasizes holistic regulation and syndrome differentiation, a specific polyherbal mixture (PM) was formulated for preweaning calves. The formulation was designed according to the TCVM theory of “Jun-Chen-Zuo-Shi” addressing the core pathogenesis of dampness-heat diarrhea, which includes dampness-heat accumulation, spleen-stomach disharmony, and dysfunctional transport [19, 20]. The therapeutic strategy was aimed at clearing heat, eliminating dampness, invigorating the spleen, and promoting digestion. The PM was composed of six medicinal herbs in equal ratios, including *Crataegus pinnatifida*, *Callicarpa nudiflora* Hook. & Arn., *Mallotus apelta *(Lour.) Müll.Arg*.*, *Amomum villosum* Lour., *Centella asiatica* (L.) Urban, and *Alpinia oxyphylla* Miq. Phytochemical analysis revealed that the active constituents of PM predominantly consisted of lignans (42.3%), flavonoids (21.2%), terpenoids (15.6%), and organic acids and their derivatives (7.8%), among others. Previous work has demonstrated that dietary supplementation with PM at 40 g/d improved weaning weight, average daily gain, and diarrhea resistance in calves [21]. However, the active components and anti-diarrheal mechanisms of PM have not been elucidated. Therefore, this study aimed to clarify the mechanisms and active constituents through which PM alleviates calf diarrhea by integrating network pharmacology, metabolomics, and 16S rRNA sequencing, thereby providing a theoretical foundation for the application of plants and their extracts in ruminants.

## Methods

### Animals, management, and experimental treatments

A total of 64 healthy Holstein calves (40 ± 4.4 kg BW; 48 females and 16 males) were selected and randomly allocated to one of four treatment groups based on birth weight, data, and sex in a completely randomized design. The study included one control group (CON) without PM supplementation and three treatment groups receiving PM at doses of 10 (PM10), 20 (PM20), or 40 (PM40) g/d per calf. The product PM was provided by Hainan Zhongmu Modern Agriculture Technology Co., Ltd. (Hainan, China). All calves were separated from dams immediately after birth and housed individually in straw-bedded pens (1.5 m × 4.0 m × 1.7 m; length × width × height). Management followed standardized protocols, feeding 4 L of high-quality colostrum (> 22% Brix) within the first hour after birth and an additional 2 L within 12 h. Calves were fed pasteurized whole milk (containing 3.79% ± 0.49% fat, 3.37% ± 0.09% CP, 4.55% ± 0.50% lactose, and 11.4% ± 0.69% TS) twice daily according to an age-dependent schedule: 6 L per meal from d 1 to 7, 8 L from d 8 to 15, 10 L from d 16 to 51, with gradual reduction until weaning at d 60. The predetermined amount of PM was added to the milk and manually stirred to ensure homogeneity before each feeding. Supplementation began at 4 days of age and continued until weaning. In addition, calves had free access to calf starter from d 4 until weaning.

### Data collection and sampling

Throughout the experimental period (d 4 to 60), fecal consistency was assessed daily by two researchers (an observer and a veterinarian) who were blinded to the treatment assignments. A standardized scoring system was applied: 1 (normal), 2 (pasty), 3 (runny), and 4 (watery). Calves with a fecal score ≥ 3 were classified as diarrheic [22]. Diarrhea frequency was calculated using the following formula: [(number of calves with diarrhea × time of diarrhea)/(total number of calves × time of trial)] × 100%. Calves exhibiting diarrhea, fever, or pneumonia were treated following the farm's standard veterinary protocols.

For hematological analysis, blood was collected from the jugular vein of 13 randomly selected calves per group into EDTA vacuum tubes on d 1, 15, 30, 45, and 60 post-morning feeding. At 60 days of age, 10 calves per group were randomly selected for comprehensive sampling. Blood was placed in additive-free tubes, followed by centrifugation at 3,500 × *g* for 15 min at 4 °C to prepare serum, which was then stored at liquid nitrogen until analysis.

At 60 days of age, 10 calves were randomly selected from each group for rumen fluid and fecal sample collection [23]. Rumen fluid samples were collected 2 h after the morning feeding using a flexible esophageal tube. After discarding the initial 50 mL to minimize saliva contamination, approximately 15 mL of rumen fluid was collected, filtered through four layers of sterile gauze, aliquoted into two 2-mL sterile nuclease-free cryovials, and immediately snap-frozen in liquid nitrogen until further analyses. Fresh fecal samples (approximately 10 g) were collected by rectal stimulation, aliquoted into two 2-mL sterile nuclease-free cryovials, and immediately snap-frozen in liquid nitrogen until further analysis.

To evaluate the development of rumen and intestinal tissues, euthanasia was performed on 16 male calves at 60 days of age. Following slaughter, tissue samples from the rumen, duodenum, jejunum, and ileum were promptly collected. The sampling sites were standardized, including the ventral sac of the rumen, duodenum (10 cm from the pylorus), jejunum (30 cm from the mesenteric junction), and ileum (10 cm anterior to the ileocecal valve) [24]. After collection, each tissue sample was gently rinsed with physiological saline to remove any residual chyme. Tissue blocks approximately 1 cm^2^ in size were then excised and fixed in 4% neutral buffered formalin for subsequent histomorphology analysis.

### Measurement of hematological parameters

Hematological parameters were analyzed using an automated blood cell analyzer (BC-30 Vet, Mindray Animal Medical Co., Ltd., Guangdong, China). The measured parameters included leukocyte, neutrophil, lymphocyte, monocyte, and erythrocyte counts; hemoglobin concentration; mean corpuscular volume (MCV), mean corpuscular hemoglobin (MCH), and mean corpuscular hemoglobin concentration (MCHC), platelet count (PLT), mean platelet volume (MPV), platelet distribution width (PDW), plateletcrit (PCT), platelet large cell count (P-LCC), and platelet large cell ratio (P-LCR).

### Histological analyses

The fixed tissue samples from the rumen, duodenum, jejunum, and ileum were subjected to graded ethanol dehydration, paraffin embedding, and sectioning. The tissue sections were deparaffinized and rehydrated, followed by staining with hematoxylin and eosin (H&E). After staining, the sections were dehydrated through a graded ethanol series, cleared with xylene, and mounted with neutral balsam. The prepared tissue sections were then observed under a microscope imaging system (SQS-600P, Shengqiang Technology Co., Ltd., Shenzhen, China). Rumen papillae height and width were measured, along with villus height and crypt depth in the duodenum, jejunum, and ileum. The villus height to crypt depth ratio (V/C) was calculated for each intestinal segment.

### Serum pharmacochemistry

Based on the fecal scores, hematological parameters, and histological analyses, serum samples from the CON and PM40 groups were selected for further analysis according to the previous publication [25]. A 100-mg sample of PM was added to 400 µL of 80% methanol solution and ground at −10 °C for 6 min. Ultrasonic extraction was then performed for 30 min, followed by a 30 min incubation at −20 °C. The extraction solution was centrifuged at 13,000 × *g* for 15 min at 4 °C, and the supernatant was collected and stored for further analysis. For blood samples of CON and PM40 groups, 100 µL was mixed with 400 µL of extraction solvent (methanol:acetonitrile = 1:1, v/v). The mixture underwent ultrasonic extraction for 30 min, followed by incubation at −20 °C for 30 min. After centrifugation at 13,000 × *g* for 15 min at 4 °C, the supernatant was collected and dried under nitrogen. The resulting residue was reconstituted in 120 µL of 50% acetonitrile, vortexed for 30 s, and subjected to a second ultrasonic extraction for 5 min. The mixture was centrifuged again at 13,000 × *g* for 10 min at 4 °C, and the supernatant was collected for analysis.

All extracts were analyzed using a UHPLC-Q Exactive system (Thermo Scientific, USA). Chromatographic separation was performed at 40 °C on an ACQUITY UPLC BEH C18 column (2.1 mm × 100 mm, 1.7 μm, Waters). The mobile phase consisted of 0.1% formic acid in 2% acetonitrile (A phase) and acetonitrile containing 0.1% formic acid (B phase), with a column flow rate of 0.4 mL/min and an injection volume of 3 µL. Mass spectrometric detection was carried out using an electrospray ionization source (ESI) in both positive and negative ion modes.

To identify the compounds absorbed into the serum of the PM40 group, the LC/MS data were processed using Progenesis QI v3.0 software. Compounds were putatively identified as potential components derived from PM based on comparison with the self-compiled Majorbio Database of Majorbio Biotechnology Co., Ltd. (Shanghai, China), MS/MS fragmentation patterns, and reference standards. Specifically, compounds present in both the PM40 group and the PM sample but absent in the CON group, and those present in PM and exhibiting a fold change (FC) > 2 between the PM40 and CON groups, were considered candidate compounds absorbed from PM.

### Network pharmacology

The potential targets for PM alleviating diarrhea were investigated following established methodologies [18, 26]. Specifically, the 22 compounds identified in the serum were considered potentially active PM constituents. The standard SMILES strings for active compounds were obtained from the PubChem database (https://pubchem.ncbi.nlm.nih.gov/). Targets of the 22 compounds were predicted using the Swiss Target Prediction online database (http://swisstargetprediction.ch). All predicted targets were then merged, and duplicates were removed to obtain all targets of PM. Disease targets associated with diarrhea were retrieved from the GeneCards database (http://www.genecards.org) using "diarrhea" as the keyword, with a relevance score cutoff > 1.42. The common targets between the PM and the diarrhea were identified using the Venny 2.1.0 (https://bioinfogp.cnb.csic.es/tools/venny/index.html). The common targets were subsequently defined as potential targets for PM in alleviating diarrhea. Subsequently, the identified common targets were converted to their corresponding bovine (*Bos taurus*) orthologs using the NCBI Ortholog Tool (https://davidbioinformatics.nih.gov/workspace.html?tool=ortholog_ws), thereby obtaining potential targets within a bovine-specific context. Furthermore, a protein–protein interaction (PPI) network was constructed using the STRING database, restricted to the species “*Bos taurus*” with a confidence score ≥ 0.7. Topological network analysis was performed using the CytoNCA plugin in Cytoscape (3.10.2), evaluating key parameters including degree centrality, betweenness centrality, eigenvector centrality, network centrality, and local average connectivity to identify core hub genes. The “ingredient–target” interaction network was constructed using the Cytoscape (3.10.2) to identify potential bioactive components. Kyoto Encyclopedia of Genes and Genomes (KEGG) pathway enrichment analysis was performed using the R software (4.4.2) to identify the mechanisms underlying potential targets.

### Molecular docking

The three-dimensional structures of the selected serum-absorbed compounds were acquired from the PubChem, while the corresponding protein crystal structures were obtained from the Protein Data Bank (PDB). Before docking, the ligand molecules were dehydrated using PyMOL software. The target proteins were prepared by adding hydrogen atoms and assigning charge distributions using AutoDock Tools. Docking simulations were conducted with AutoDock Vina, with the grid box centered on the predicted active site of each receptor. The resulting binding conformations were analyzed for their binding energies (kcal/mol) and visualized through PyMOL for three-dimensional representation and LigPlot + for detailed two-dimensional interaction diagrams.

### Untargeted metabolomics analyses

Serum samples previously utilized for the serum pharmacochemistry assay were selected for untargeted metabolomic profiling. Sample preparation, liquid phase, and mass spectrometry parameters were the same as those of the serum pharmacochemistry experiments. The pretreatment of LC/MS raw data was performed by Progenesis QI v3.0 software, and the obtained MS and MS/MS mass spectral information was matched against the HMDB (http://www.hmdb.ca/), Metlin (https://metlin.scripps.edu/), and the self-compiled Majorbio Database of Majorbio Biotechnology Co., Ltd. (Shanghai, China). Data processing and analysis were conducted on the MajorBio Cloud Platform (cloud.majorbio.com). Orthogonal partial least-squares discriminant analysis (OPLS-DA) analysis was performed on the metabolomics data, and the validity of this model was assessed using the model parameters R^2^X, R^2^Y, and Q^2^. Differential metabolites were screened using the Wilcoxon rank-sum test. The screening criteria were defined as follows: *P* < 0.05 (FDR correction), VIP > 1, and |FC| > 1. Based on differential metabolites, pathway enrichment analysis was performed using the KEGG pathway database [27]. Linear regression analysis was conducted between diarrhea frequency and the top 30 differential metabolites, using R^2^ > 0.5 and *P* < 0.05 as the criteria to identify metabolites significantly correlated with diarrhea frequency.

### 16S rRNA sequencing and analysis

According to the protocols of manufacture, DNA from rumen fluid and fecal samples was extracted using the MagAtrract PowerSoil Pro DNA Kit (Qiagen, Hilden, Germany). The bacterial 16S rRNA gene was amplified using universal bacterial primers 27 F and 1492 R. All amplicons were sequenced following the standard protocols on the PacBio Sequel IIe System (Pacific Biosciences, CA, USA). Bioinformatic analysis and visualization of the rumen fluid and fecal microbiota were carried out using the Majorbio Cloud platform (www.majorbio.com). The α-diversity (including Chao1, Shannon, ACE, and Simpson indices) between groups was evaluated using the Wilcoxon rank-sum test at the ASV level. Principal coordinate analysis (PCoA) was performed based on the Bray–Curtis distance, and the significance of differences in microbial community structure between groups was assessed using ADONIS. Linear discriminant analysis effect size (LEfSe) was employed to identify microbial taxa with significant differences between groups at the species level. Differential analysis was performed on species—level microorganisms with a prevalence ≥ 10% and an average relative abundance ≥ 0.01% across all samples by the Wilcoxon rank-sum test and FDR correction.

### Statistical analyses

All data were analyzed using the UNIVARIATE procedure in SAS (version 9.4, SAS Institute Inc.) to assess homogeneity of variances and normality. Non-normally distributed data were transformed to conform to a normal distribution. Models for the occurrence of diarrhea (≥ 3) were evaluated using the PROC GLIMMIX with logistic distribution. The odds ratios were used to compare the likelihood of diarrhea occurring in calves in each treatment group. Hematological parameters were analyzed using the MIXED procedures. The model included PM treatment, covariate, time and the interaction between treatment and time. Time was specified as a repeated measure, calf as subject, and birth hematological parameters as a covariate, using a compound symmetry structure. Gastrointestinal morphometrics data were analyzed via one-way ANOVA. Statistical significance was defined as *P* < 0.05, while a statistical trend was defined as 0.05 ≤ *P* < 0.10. A Spearman's rank correlation analysis was conducted using R software.

## Results

### Diarrhea

Table 1 and Fig. S1 show the logistic models for calf diarrhea (≥ 3). The probability of diarrhea occurrence was significantly reduced in the PM40 group calves than in the CON group calves (odds ratio = 0.69; *P* = 0.01).

**Table 1:** Logistic model for the frequency of diarrhea in Holstein calves as influenced by dietary supplements without (CON) or with varying doses of polyherbal mixtures (PM10 = 10, PM20 = 20, PM40 = 40 g/d) during the experimental period (d 4–60) (*n* = 16 per treatment)

Variable and comparison	Coefficient	SEM	OR	95% CI	*P*-value
PM10 vs. CON	−0.0893	0.14	0.91	0.69, 1.21	0.53
PM20 vs. CON	−0.1850	0.14	0.83	0.63, 1.10	0.20
PM40 vs. CON	−0.3752	0.15	0.69	0.51, 0.92	0.01

### Hematological parameters

As presented in Table 2, lymphocytes, erythrocytes, hemoglobin, MCV, MCH, MCHC, PLT, MPV, PDW, P-LCC concentrations, and the percentage of neutrophil, lymphocyte, monocyte, hematocrit, PCT, and P-LCR were not affected by PM addition (*P* > 0.05). However, leukocyte concentration showed marginal significance (*P* = 0.05), with higher levels in the PM40 group. Neutrophil concentration showed marginally significant interactions between treatment and time (*P* = 0.07), with higher levels in the PM40 group. Monocyte concentration was significantly elevated in the PM40 group than in the CON group (*P* < 0.01).

**Table 2:** Effects of polyherbal mixtures (PM) on blood hematological parameters in preweaning calves (*n* = 13 per treatment)

**Items**^**1**^	**Treatment**^**2**^				**SEM**	**Effects**^**3**^** (*P*-value)**		
	**CON**	**PM 10**	**PM 20**	**PM 40**		**T**	**Time**	**T × Time**
Leukocyte, 10^9^/L	9.64	9.88	9.62	10.7	0.17	0.05	< 0.01	0.17
Neutrophil, 10^9^/L	5.31	5.50	5.09	5.74	0.15	0.36	< 0.01	0.07
Lymphocyte, 10^9^/L	4.13	4.06	4.13	4.23	0.08	0.85	< 0.01	0.64
Monocyte, 10^9^/L	0.53^b^	0.55^b^	0.53^b^	0.62^a^	0.01	< 0.01	< 0.01	0.59
Erythrocyte, 10^12^/L	9.07	9.30	9.16	9.00	0.09	0.61	< 0.01	0.29
Hemoglobin, g/L	106	109	106	104	1.01	0.54	< 0.01	0.22
Neutrophil, %	52.0	53.5	49.9	52.0	0.70	0.12	< 0.01	0.51
Lymphocyte, %	42.5	41.1	44.0	42.4	0.68	0.22	< 0.01	0.58
Monocyte, %	5.54	5.65	5.57	5.53	0.04	0.79	0.05	0.76
Hematocrit, %	36.0	36.3	35.9	35.5	0.35	0.90	< 0.01	0.55
MCV, fL	39.6	39.2	40.0	39.3	0.29	0.62	< 0.01	0.92
MCH, pg	11.8	11.6	11.8	11.6	0.08	0.63	< 0.01	0.97
MCHC, g/L	296	297	295	294	0.62	0.17	< 0.01	0.42
PLT, 10^9^/L	907	815	893	906	19.2	0.19	< 0.01	0.72
MPV, fL	5.39	5.37	5.40	5.36	0.02	0.96	< 0.01	0.45
PDW, %	14.3	14.2	14.3	14.2	0.02	0.48	< 0.01	0.67
PCT, %	0.50	0.44	0.47	0.48	0.01	0.39	< 0.01	0.68
P-LCC, 10^9^/L	197	163	191	195	6.92	0.16	< 0.01	0.67
P-LCR, %	20.7	20.5	20.9	20.3	0.28	0.92	< 0.01	0.71

^1^*MCV *Mean corpuscular volume, *MCH *Mean corpuscular hemoglobin, *MCHC *Mean corpuscular hemoglobin concentration, *PLT *Platelet, *MPV *Mean platelet volume, *PDW *Platelet distribution width, *PCT *Plateletcrit, *P-LCC *Platelet large cell count, *P-LCR *Platelet large cell ratio

^2^CON = control, supplemented with no PM; PM10 = 10 g/d PM supplemented diet; PM20 = 20 g/d PM supplemented diet; PM40 = 40 g/d PM supplemented diet

^3^T = treatment effects; T × Time = interaction between treatment and time

^a,b ^Means within a row with different superscripts are significant differences (*P* < 0.05)

### Morphometric assessments of the gastrointestinal tract

**Fig. 1:**
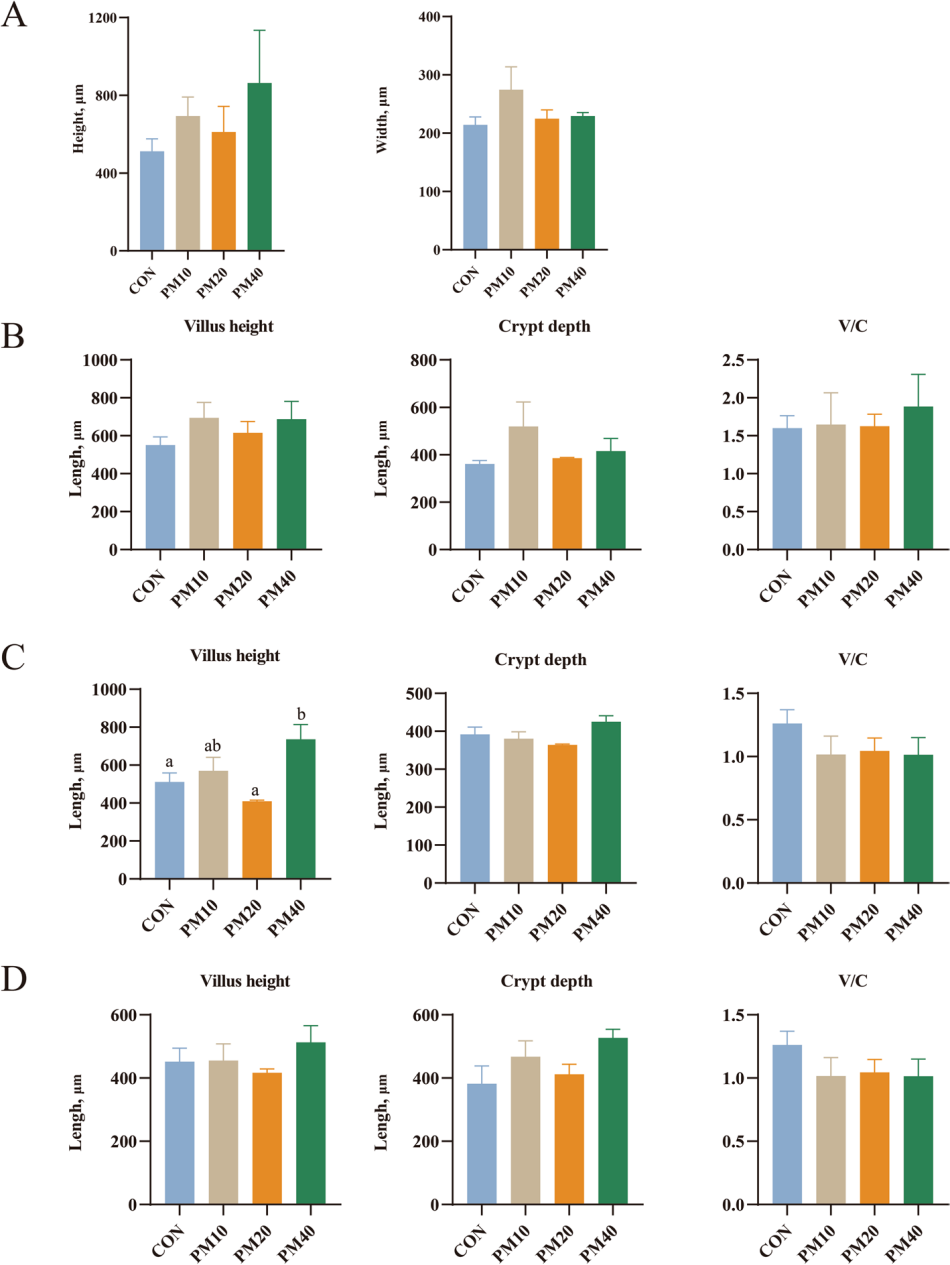
Effects of supplementing with polyherbal mixtures (PM) on the development of rumen papilla, duodenum, jejunum, and ileum on d 60 in Holstein calves. **A** Quantitative analysis of rumen papilla height and width stained with H&E (*n* = 4). **B** Quantitative analysis of duodenum stained with H&E (*n* = 4). **C** Quantitative analysis of jejunum stained with H&E (*n* = 4). **D** Quantitative analysis of ileum stained with H&E (*n* = 4). CON = control, supplemented with no PM; PM10 = 10 g/d PM supplemented diet; PM20 = 20 g/d PM supplemented diet; PM40 = 40 g/d PM supplemented diet. Data are shown as LSM ± SEM

Figure 1 shows the results of the evaluation of the height and width of the rumen papillae and the villus height and crypt depth of the duodenum, jejunum, and ileum on 60 d old calves. No significant effects of PM supplementation were observed on rumen papilla height or width (*P* > 0.05). Similarly, no differences were detected among groups in duodenal villus height and crypt depth, jejunal crypt depth, ileal villus height and crypt depth, or the V/C in any intestinal segment (*P* > 0.05). However, jejunal villus height was significantly greater in PM40 group than in CON group (*P* = 0.01).

### Analysis of components absorbed into the blood of PM-treated calves

**Fig. 2:**
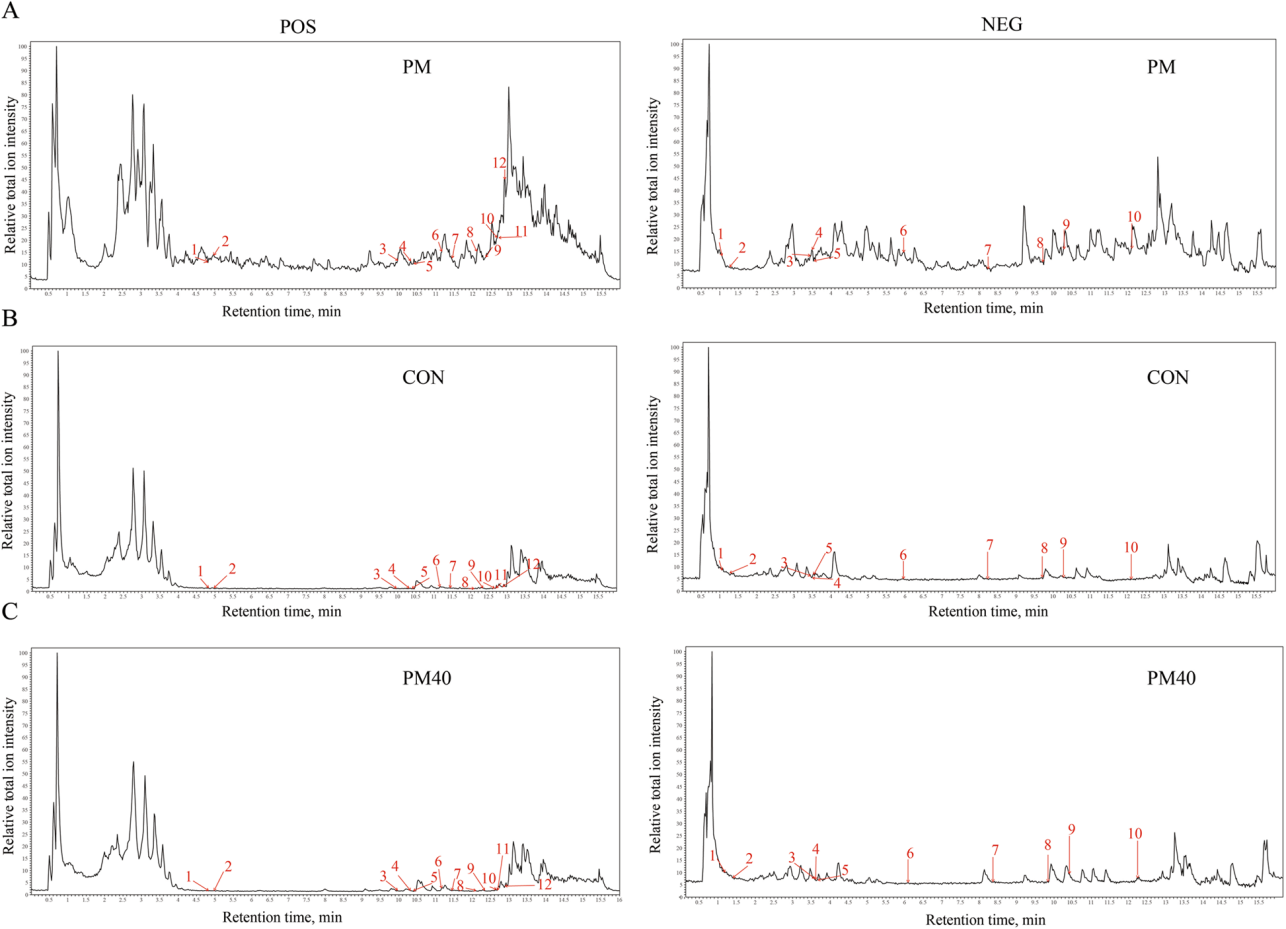
Total ion chromatograms. **A** Base peak chromatogram of PM in positive ion mode and negative ion mode. **B** Baseline chromatograms of control serum in positivTe ion mode and negative ion mode. **C** Base peak chromatogram of PM40 group serum in positive ion mode and negative ion mode

Pharmaceutical chemical analysis of the aqueous PM extract identified a total of 427 chemical constituents, including 85 flavonoids, 79 terpenoids, 33 organic acids and derivatives, 20 coumarins and derivatives, 18 carbohydrates, 13 amino acids and analogues, 13 lignans, 11 phenols, 11 sterols and derivatives, 6 quinonoids, and 138 other compounds. Comparative analysis with serum from the CON group revealed that calves in the PM40 group absorbed 22 prototypical compounds in their blood, including 12 terpenoids, 5 flavonoids, 3 organic acids, 1 carbohydrate, and 1 polyphenol (Fig. 2, Table 3).

**Table 3:** Identification of absorbed phytochemical components from polyherbal mixtures in calf serum

No.^a^	Retention time, min	Formula	*m/z*	Mass error, ppm	Identification
Mol 1	5.96	C_13_H_8_O_4_	227.03	−2.50	3,8-dihydroxy-urolithin
Mol 2	10.32	C_16_H_12_O_5_	285.08	−2.29	Acacetin
Mol 3	12.10	C_20_H_30_O_5_	349.20	−0.61	Andrographolide
Mol 4	1.06	C_12_H_16_O_7_	271.08	−0.09	Arbutin
Mol 5	4.83	C_15_H_22_O_5_	283.15	−2.26	Artemisinin
Mol 6	10.38	C_15_H_20_O_2_	233.15	−2.01	Atractylenolide II
Mol 7	3.46	C_15_H_22_O_9_	345.12	−0.41	Aucubin
Mol 8	12.34	C_20_H_28_O_3_	317.21	−2.42	Cafestol
Mol 9	9.97	C_15_H_10_O_4_	255.06	−2.50	Chrysin
Mol 10	10.28	C_15_H_24_O_2_	235.17	−2.27	Dihydroartemisinic acid
Mol 11	12.86	C_15_H_22_O	201.16	−1.45	Germacrone
Mol 12	12.66	C_20_H_28_O_5_	331.19	−2.53	Ingenol
Mol 13	11.43	C_20_H_26_O_3_	297.18	−2.19	Kahweol
Mol 14	4.96	C_15_H_16_O_4_	261.11	−2.77	Linderane
Mol 15	8.23	C_15_H_14_O_4_	257.08	−1.73	Lunularic acid
Mol 16	9.70	C_18_H_16_O_7_	343.08	−0.17	Lysionotin
Mol 17	1.26	C_7_H_10_O_5_	173.04	−5.52	Shikimic acid
Mol 18	12.11	C_20_H_30_O_3_	319.23	−2.47	Steviol
Mol 19	3.52	C_9_H_10_O_5_	197.04	−3.99	Syringic acid
Mol 20	12.68	C_16_H_12_O_4_	269.08	−2.34	Tectochrysin
Mol 21	11.16	C_20_H_24_O_3_	313.18	−2.40	Triptophenolide
Mol 22	3.48	C_27_H_30_O_15_	593.15	0.73	Vicenin II

^a^Serum samples were collected from calves at 60 days of age

### Network pharmacology analysis

**Fig. 3:**
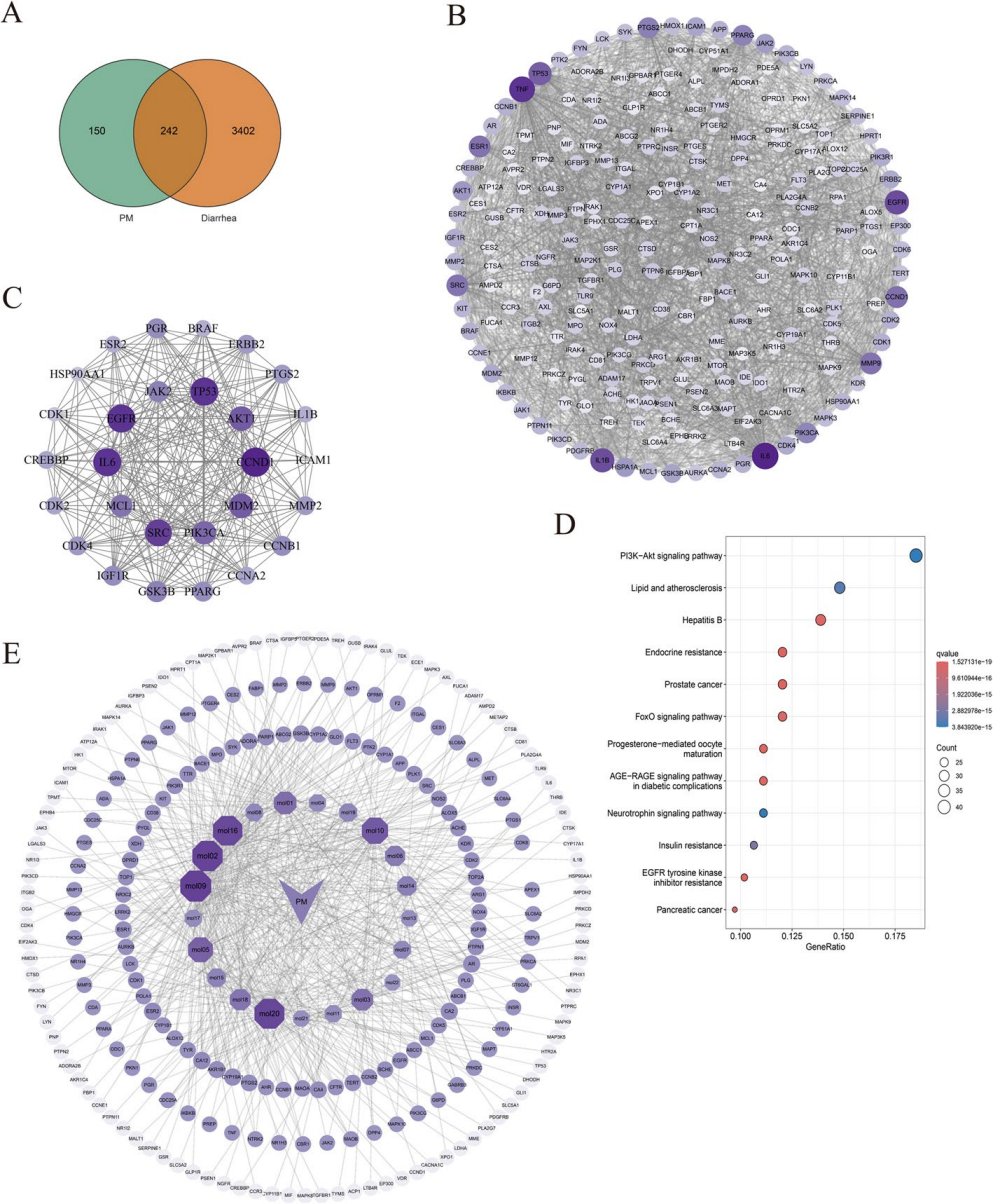
Network pharmacology analysis predicts potential targets and signaling pathways of PM in alleviating calf diarrhea. **A** PM – Diarrhea target Venn diagram. **B** PPI network of intersection targets. **C** Subnet after core target screening. **D** KEGG pathway analysis bubble chart for the top 12 signaling pathways. **E** “Ingredient – Target” Network. Mol02 = Acacetin; Mol09 = Chrysin; Mol10 = Dihydroartemisinic acid; Mol16 = Lysionotin; Mol20 = Tectochrysin

A total of 150 gene targets associated with PM blood components and 3,402 diarrhea-related targets were identified. Intersection analysis revealed 242 common targets (Fig. 3A), of which 225 were successfully converted to bovine orthologs. From the subsequent PPI network of these 225 targets (Fig. 3B), 28 core target proteins were identified through topological analysis, with selection criteria requiring that degree centrality (DC), betweenness centrality (BC), eigenvector centrality (EC), network centrality (NC), and local average centrality (LAC) values all exceeded their respective median thresholds (Fig. 3C and Table 4). Functional enrichment analysis via KEGG revealed 177 significantly enriched pathways. Figure 3D shows the top 12 pathways, with the PI3K/AKT signaling pathway being prioritized for further investigation. The “component-target-disease” network was visualized using Cytoscape software. Through network topology analysis, five core components were identified, including acacetin, chrysin, tectochrysin, dihydroartemisinic acid, and lysionotin, comprising 5 flavonoid compounds and 1 terpenoid compound (Fig. 3E).

**Table 4:** Topological characteristics of the core targets in the PPI network^a^

Target	DC	BC	EC	NC	LAC
CCND1	27.0	20.6	0.2	27.0	18.1
TP53	26.0	15.3	0.2	25.5	18.2
IL6	26.0	17.8	0.2	25.3	17.8
EGFR	26.0	19.0	0.2	25.1	17.5
SRC	25.0	15.5	0.2	23.7	17.4
AKT1	23.0	12.7	0.2	20.4	16.1
MDM2	23.0	9.6	0.2	21.0	16.9
PIK3CA	22.0	8.4	0.2	19.9	16.3
MCL1	21.0	7.9	0.2	18.1	15.5
JAK2	20.0	8.4	0.2	16.9	14.6
GSK3B	20.0	6.1	0.2	17.1	15.2
PGR	19.0	6.4	0.2	15.7	14.0
IGF1R	19.0	4.4	0.2	16.2	14.9
CCNB1	19.0	4.8	0.2	16.2	14.7
PPARG	19.0	7.2	0.2	15.8	13.9
PTGS2	18.0	5.6	0.2	15.2	13.7
MMP2	18.0	5.6	0.2	15.0	13.6
ERBB2	18.0	4.9	0.2	15.1	13.8
CDK4	18.0	5.5	0.2	14.2	13.1
CREBBP	17.0	4.3	0.2	14.1	13.1
CCNA2	17.0	2.8	0.2	14.8	13.8
CDK2	17.0	5.2	0.2	13.4	12.4
IL1B	16.0	3.1	0.2	14.1	12.9
ESR2	16.0	3.1	0.2	13.7	12.8
BRAF	16.0	2.4	0.2	14.0	13.0
HSP90AA1	14.0	3.4	0.1	10.8	10.0
CDK1	14.0	1.9	0.1	12.2	11.3
ICAM1	10.0	0.0	0.1	10.0	9.0

^a^*DC *Degree centrality, *BC *Betweenness centrality, *EC *Eigenvector centrality, *NC *Network centrality, *LAC *Local average centrality

### Molecular docking

**Fig. 4:**
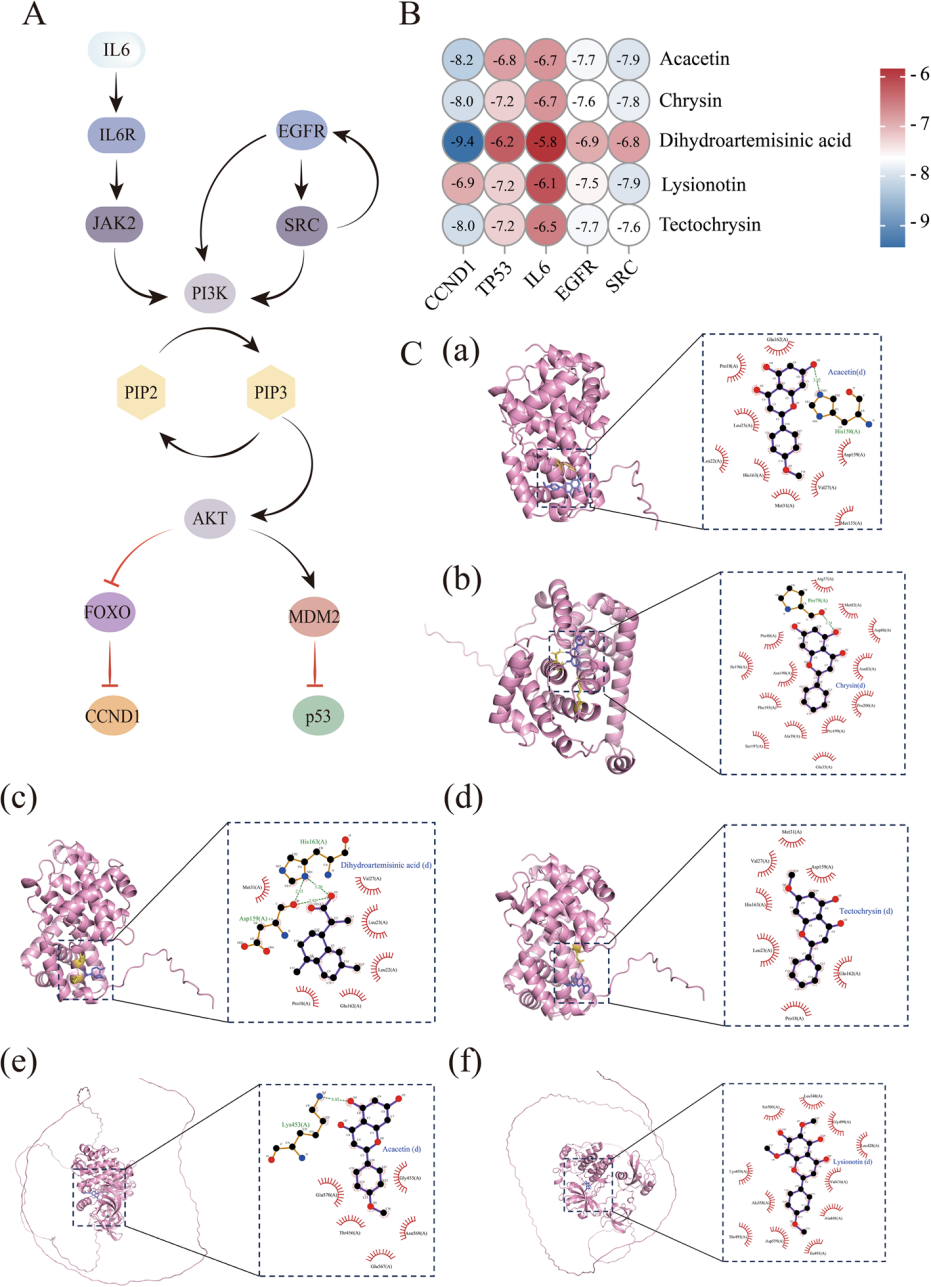
Molecular docking validation of active components in PM against the PI3K/AKT signaling pathway. **A** Interaction network among key regulators (CCND1, TP53, IL6, EGFR and SRC) in the PI3K/AKT signaling pathway. **B** Binding affinities of five core target proteins with five key components in PM. **C** 3D and 2D schematic diagrams of optimal docking results: (**a**) Acacetin – CCND1, (**b**) Chrysin – CCND1, (**c**) Dihydroartemisinic acid – CCND1, (**d**) Tectochrysin – CCDN1, (**e**) Acacetin – SRC, (**f**) Lysionotin – SRC

Among the core target proteins identified, the top five targets (CCND1, TP53, IL6, EGFR, and SRC) ranked by DC value were selected as key regulators of the PI3K/AKT signaling pathway for further investigation (Fig. 4A). Molecular docking was performed to evaluate the binding interactions between these five target proteins and the five core PM components. The results demonstrated strong binding affinity between the target proteins and PM components, with all docking scores below −5 kcal/mol (Fig. 4B). Representative docking poses with optimal binding configurations were further visualized through three-dimensional and two-dimensional interaction diagrams (Fig. 4C).

### Serum metabolic profiling

**Fig. 5:**
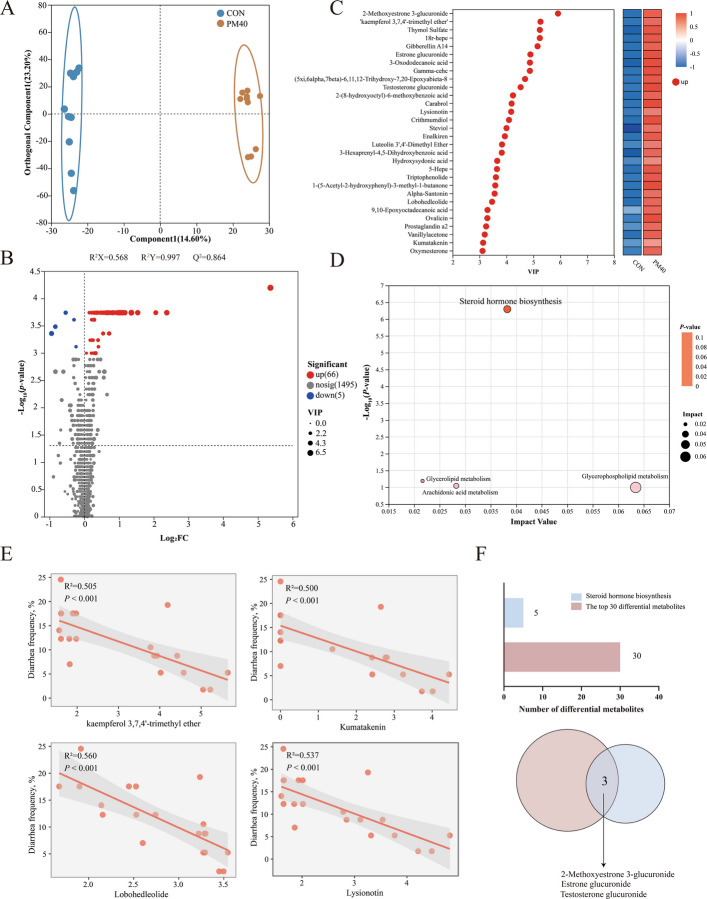
Polyherbal mixtures (PM) supplementation altered the serum metabolic profile in preweaning calves (*n* = 10). **A** Orthogonal partial least squares discriminant analysis (OPLS-DA) scores of serum metabolites in CON and PM40 groups in mixed mode. **B** The serum differential metabolites as affected by PM40. **C** The top 30 serum differential metabolites by KEGG compound database [CON/PM40, variable importance in the projection (VIP > 3), *P* < 0.05]. **D** Metabolic pathways were analyzed based on different metabolites. **E** Linear correlation analysis between diarrhea frequency and kaempferol 3,7,4'-trimethyl ether, kumatakenin, lobohedleolide, and lysionotin. **F** The Venn diagram illustrates the common differential metabolites among the top 30 differential metabolites and those enriched in the steroid biosynthesis pathway. CON = control, supplemented with no PM; PM40 = 40 g/d PM supplemented diet

OPLS-DA revealed distinct metabolite profiles between CON and PM40 groups (R^2^X = 0.568, R^2^Y = 0.997, Q^2^ = 0.864) (Fig. 5A). In mix mode, 66 metabolites were significantly upregulation and 5 metabolites were significantly downregulation in the PM40 group compared with the CON group (Fig. 5B). Based on a combination of statistical analysis and VIP values derived from the OPLS-DA model, the top 30 differential metabolites were identified, as illustrated in Fig. 5C. KEGG pathway analysis revealed that the differential metabolites were mainly enriched in steroid hormone biosynthesis (Fig. 5D). Linear regression analysis revealed that kaempferol 3,7,4'-trimethyl ether, kumatakenin, lobohedleolide and lysionotin were the metabolites most significantly correlated with diarrhea frequency (R^2^ > 0.5, *P* < 0.05, Fig. 5E). 2-Methoxyestrone 3-glucuronide, estrone glucuronide, and testosterone glucuronide were identified as differential metabolites within the steroid hormone biosynthesis and were also among the top 30 differential metabolites.

### Rumen microbiota

**Fig. 6:**
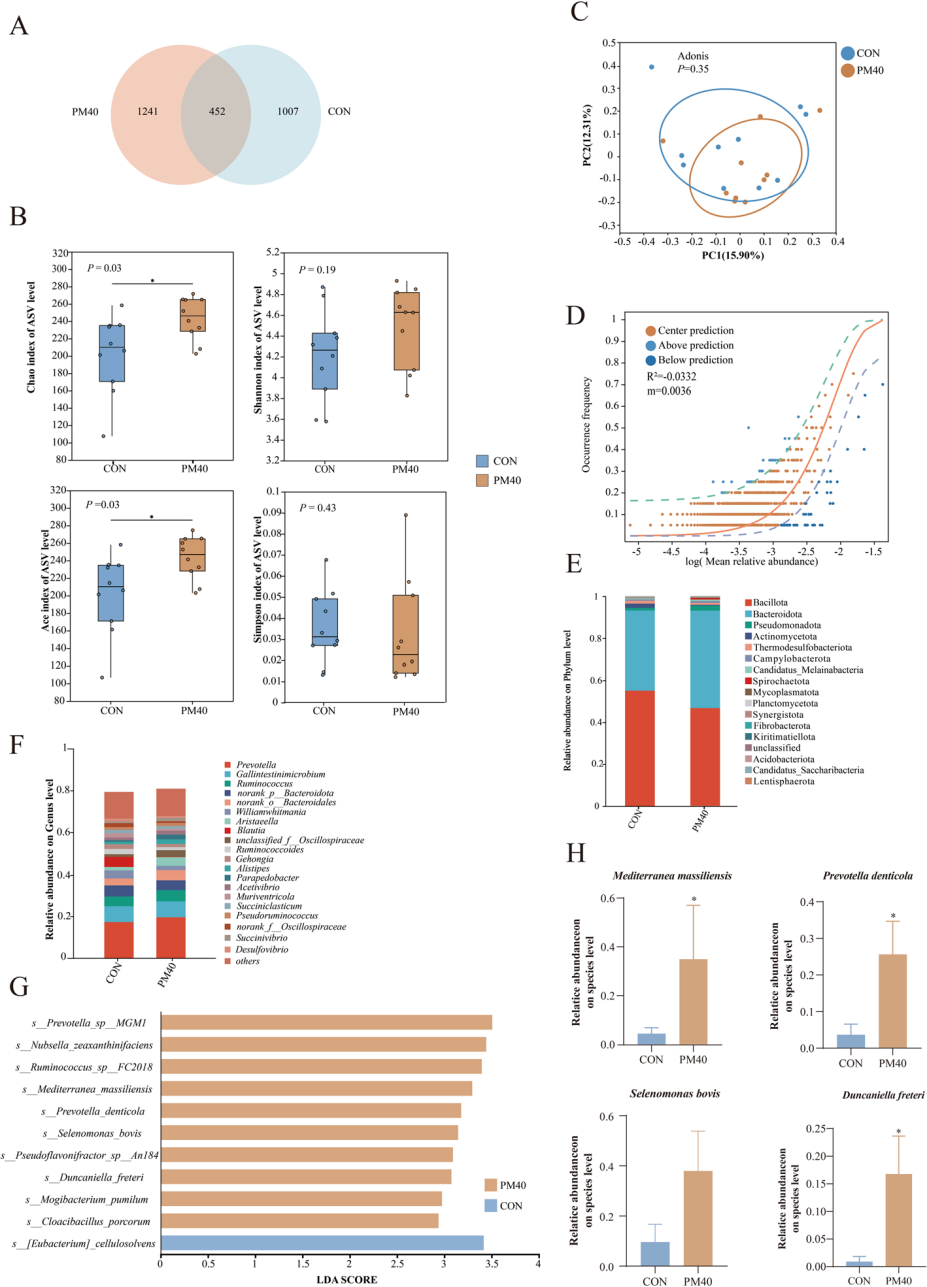
PM regulated the diversity and composition of rumen microbiota in 60 d old calves (*n* = 10). **A** Visualize the Venn diagram of common and unique ASVs. **B** Community diversity and richness. **C** PCOA analysis. **D** NCM analysis. **E** Microbial composition at the phylum level. **F** Microbial composition at the genus level. **G** Histogram of LEfSe analysis; CON = control, supplemented with no PM; PM40 = 40 g/d PM supplemented diet. **H** Relative proportion of microbiota on Species. All data were represented as mean ± SEM. ^*^P < 0.05

As shown in Fig. 6A, 452 ASVs were shared between the CON and the PM40 groups, while 1,007 ASVs and 1,241 ASVs were unique to the CON and PM40 groups, respectively. At 60 days of age, the Chao and ACE indices in PM40 group were significantly greater than in CON group (*P* < 0.05) (Fig. 6B). OPLS-DA revealed no significant differences in rumen microbial communities between the two groups (Fig. 6C). Based on neutral community model analysis, the lower R^2^ value showed that rumen microbial community construction was primarily driven by deterministic processes under PM treatment, with random processes playing a minor role (Fig. 6D). As shown in Fig. 6E and F, rumen microorganisms were mainly classified into the Bacillota, Bacteroidota, Pseudomonadota, and Actinomycetota at the phylum level, and the *Prevotella*, *Gallintestinimicrobium*, *Ruminococcus*, and *norank-p-Bacteroidota* at the genus level. LEfSe revealed significant differences in microbial communities among the CON and the PM40 groups (Fig. 6G). Specifically, at the species level, PM treatment significantly increased *Mediterranea massiliensis*, *Prevotella denticola,* and *Duncaniella freteri* relative abundance (*P* < 0.05; Fig. 6H).

### Fecal microbiota

**Fig. 7:**
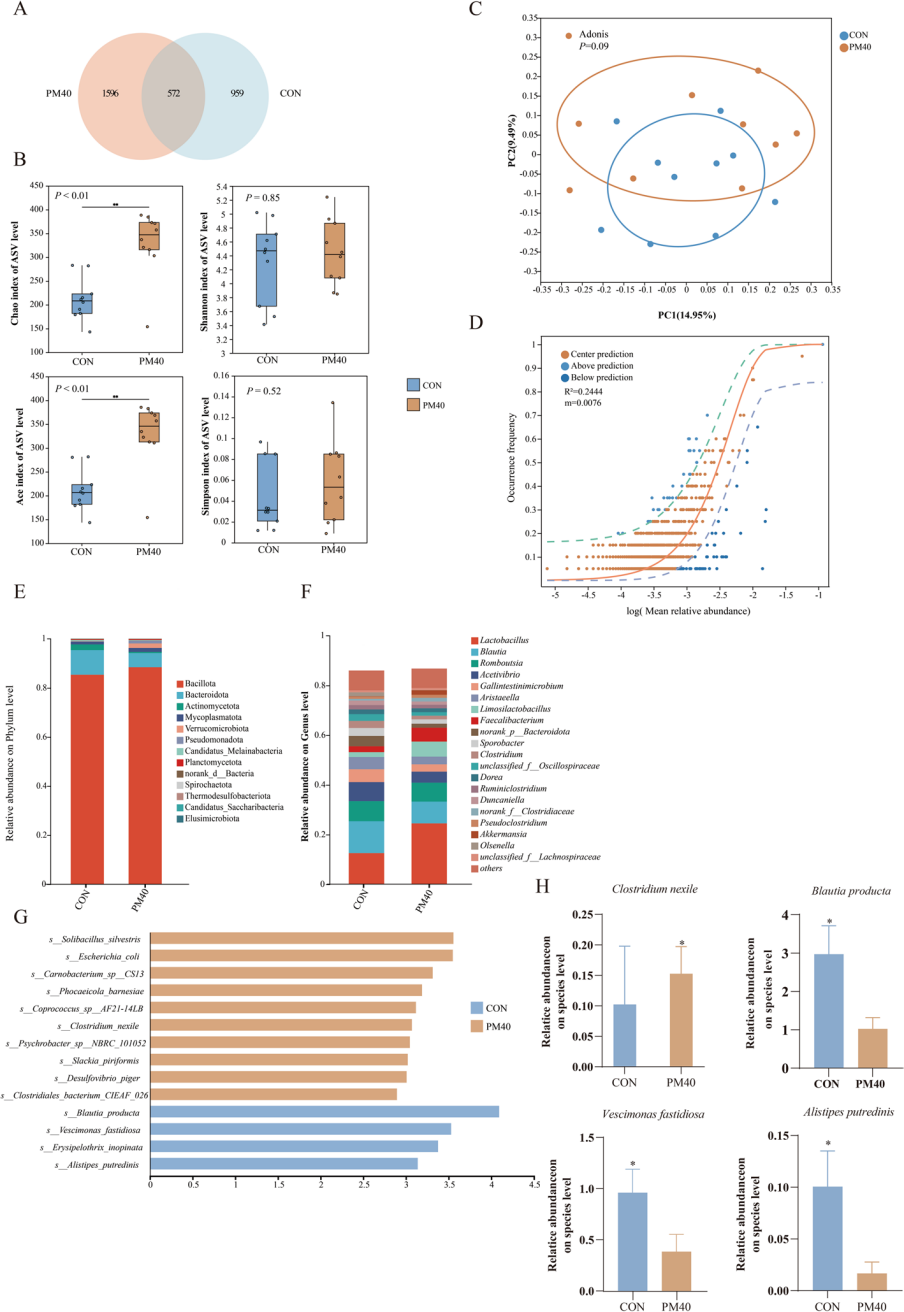
PM regulated the diversity and composition of fecal microbiota in 60 days old calves (*n* = 10). **A** Visualize the Venn diagram of common and unique ASVs. **B** Community diversity and richness. **C** PCOA analysis. **D** NCM analysis. **E** Microbial composition at the phylum level. **F** Microbial composition at the genus level. **G** Histogram of LEfSe analysis; CON = control, supplemented with no PM; PM40 = 40 g/d PM supplemented diet. **H** Relative proportion of microbiota on Species. All data were represented as mean ± SEM. ^*^P < 0.05

A similar pattern was observed in the cecal microbiota, with 572 ASVs shared between the CON and PM40 groups. In comparison, 959 and 1,596 ASVs were unique to the CON and PM40 groups, respectively (Fig. 7A). At 60 days of age, the Chao and ACE indices were significantly greater in PM40 group than in CON group (*P* < 0.05; Fig. 7B). OPLS-DA model revealed a trend toward separation among the CON and PM40 groups (*P* = 0.09; Fig. 7C). Combined with neutral community model analysis, the lower R^2^ value indicated that the construction of the cecal microbiota was more driven by the deterministic selection effect of PM treatment, while the influence of random processes was relatively weak (Fig. 7D). As shown in Fig. 7E and F, the cecal microbiota with higher abundance at the phylum level were Bacillota, Bacteroidota, Actinomycetota, and Mycoplasmatota, while at the genus level, *Lactobacillus*, *Blautia*, *Romboutsia*, and *Acetivibrio* were predominant. LEfSe confirmed significant differences in microbial communities among the two groups (Fig. 7G). At the species level, *Clostridium nexile* relative abundance significantly increased in the PM40 group than in the CON group, whereas *Blautia producta*, *Vescimonas fastidiosa*, and *Alistipes putredinis* relative abundance significantly decreased (*P* < 0.05; Fig. 7H).

### Correlations between active ingredients absorbed in blood and metabolites

**Fig. 8:**
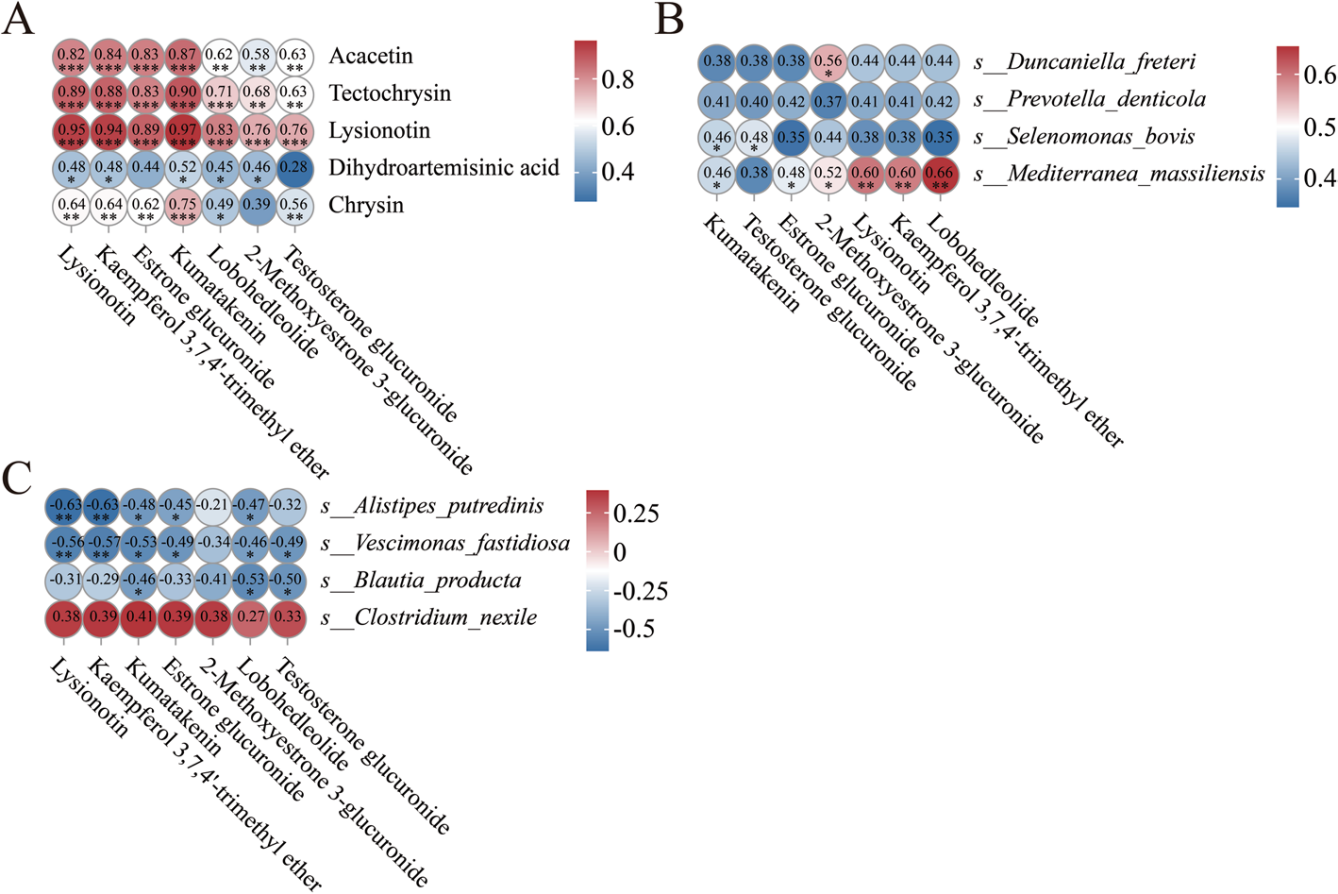
Relationships of serum key metabolites with active components in PM and with key bacteria in the rumen and feces. **A** Heatmaps of Spearman correlations between the selected active ingredients absorbed in blood and the selected differential metabolites. **B** Heatmaps of Spearman correlations between the selected differential metabolites and rumen microbiota. **C** Heatmaps of Spearman correlations between the selected differential metabolites and fecal microbiota. ^*^P < 0.05, ^**^P < 0.01, ^***^P < 0.001

Spearman correlation analysis revealed relationship between the selected absorbed bioactive components and the selected differential metabolites (Fig. 8A). Acacetin, tectochrysin, lysionotin were significant positively correlated with lysionotin, kaempferol 3,7,4'-trimethyl ether, estrone glucuronide, kumatakenin, lobohedleolide, 2-Methoxyestrone 3-glucuronide and testosterone glucuronide (*P* < 0.05). Dihydroartemisinic acid exhibited significant correlations with lysionotin, kaempferol 3,7,4'-trimethyl ether, kumatakenin, lobohedleolide and 2-Methoxyestrone 3-glucuronide (*P* < 0.05). Similarly, chrysin exhibited a significant positive correlation with lysionotin, kaempferol 3,7,4'-trimethyl ether, estrone glucuronide, kumatakenin, lobohedleolide, and testosterone glucuronide (*P* < 0.05).

### Correlations between microbes and metabolites

Spearman correlation analysis revealed a significant positive correlation between *Duncaniella freteri* and 2-methoxyestrone 3-glucuronide (*P* < 0.05). *Selenomonas bovis* was positively correlated with kumatakenin and testosterone glucuronide (*P* < 0.05). *Mediterranea massiliensis* exhibited a significant positive correlation with kumatakenin, estrone glucuronide, 2-Methoxyestrone 3-glucuronide, lysionotin, kaempferol 3,7,4'-trimethyl ether, and lobohedleolide (*P* < 0.05, Fig. 8B).

Similarly, *Alistipes putredinis* and *Vescimonas fastidiosa* exhibited negative correlations with lysionotin, kaempferol 3,7,4'-trimethyl ether, kumatakenin, estrone glucuronide, lobohedleolide and testosterone glucuronide (*P* < 0.05). *Blautia producta* exhibited negative correlations with kumatakenin, lobohedleolide, and testosterone glucuronide (*P* < 0.05, Fig. 8C).

## Discussion

It is well-established that plants and their extracts can be utilized as feed additives, growth promoters, and immune enhancers to improve animal health and production performance [28]. Previous studies have shown that the addition of PM can effectively increase the average daily gain and reduce the diarrhea frequency in preweaning calves [21]. However, its underlying anti-diarrheal mechanisms and pharmacodynamic material basis remain incompletely elucidated. The present study demonstrates that daily supplementation with 40 g/d of PM effectively alleviated calf diarrhea by modulating serum steroid hormone biosynthesis and enhancing microbial diversity in both rumen and feces. Acacetin, chrysin, tectochrysin, dihydroartemisinic acid, and lysionotin may serve as potential active constituents.

Hematological parameters are widely used to assess health status [29]. All measured serum biochemical values in this trial remained within normal physiological ranges [30]. Leukocyte and monocyte concentrations were higher in the PM40 group than in the CON group, which may indicate enhanced immune homeostasis and mucosal surveillance capabilities [31]. Villus height and crypt depth represent key morphological indicators for evaluating intestinal health [32]. Diarrhea typically induces intestinal dysfunction characterized by villus atrophy and crypt deepening [33]. This morphological adaptation enlarges the absorptive surface area of the jejunal mucosa, thereby promoting water and electrolyte reabsorption and reducing the risk of osmotic diarrhea [34]. Furthermore, in conjunction with the reduced incidence of diarrhea and elevated concentrations of leukocytes and monocytes observed in the PM40 group, the increased villus height may reflect ongoing intestinal mucosal repair and maturation processes [35].

A compound-target-pathway interaction network was constructed based on the 22 systemically absorbed compounds identified in this study. PPI network topological analysis revealed SRC, CCND1, EGFR, IL6, and TP53 as core targets, while KEGG enrichment analysis indicated the potential significance of the PI3K/AKT signaling pathway. Based on network pharmacology analysis, acacetin, chrysin, tectochrysin, dihydroartemisinic acid, and lysionotin were identified as potential key active constituents in PM. These compounds, primarily classified as flavonoids and terpenoids, have documented anti-inflammatory [36], antioxidant [37], and epithelial-protective activities [38], suggesting that they may contribute to the beneficial effects of PM on intestinal health.

In this study, the core targets identified are functionally associated with the maintenance of epithelial homeostasis, inflammatory regulation, stress responses, and cell cycle control [39, 40]. Furthermore, the PI3K/AKT signaling pathway has been widely demonstrated to be involved in epithelial survival, mucosal repair, and the maintenance of barrier function [41]. Molecular docking analysis revealed strong binding affinities (binding energy < −5 kcal/mol) between the core targets (EGFR, SRC, IL6, CCND1, and TP53) and these putative active constituents. These findings provide supportive computational evidence for potential compound-target interactions. However, it is important to note that network pharmacology and molecular docking are primarily predictive methods and not direct experimental validations. Furthermore, in this study, candidate targets predicted from human databases were converted to bovine genes through NCBI ortholog tool. This strategy is biologically reasonable because orthologous genes originate from a common ancestral gene and often retain conserved molecular functions across species [42]. Furthermore, comparative transcriptomic analysis revealed that homologous genes between humans and cattle generally exhibited conserved expression patterns across immune tissues [43]. However, orthologous relationships do not necessarilly imply complete functional equivalence, as species–species divergence in gene regulation, expression context, and downstream interraction networks may still occur [42]. Thus, the network pharmacology findings in this study should be considered as hypothesis-generating evidence. Further direct experimental validation of key molecules within the PI3K/AKT pathway in bovine intestinal tissue is warranted to confirm the proposed hypothesis.

Serum metabolites are crucial indicators of systemic metabolic status and physiological health [44]. In this study, the PM40 group calves exhibited significantly higher serum levels of flavonoids and terpenoids, primarily including kaempferol 3,7,4'-trimethyl ether, kumatakenin, lobohedleolide, and lysionotin. Kaempferol 3,7,4'-trimethyl ether is a flavonol aglycone derived from kaempferol and has been reported to possess antioxidant activity [45]. Chen et al. [46] and Shazia et al. [47] reviewed that kaempferol and its methylated derivatives suppress the activation of the NF-κB and MAPK pathways, thereby downregulating the expression of pro-inflammatory cytokines such as TNF-α, IL-6, and IL-1β. Kumatakenin is a flavonoid compound. In a DSS-induced colitis mouse model, administration of kumatakenin at doses of 25 mg/kg and 100 mg/kg significantly reduced colonic levels of TNF-α and IL-6, decreased myeloperoxidase activity, and lowered serum fluorescein isothiocyanate-dextran levels [48]. Lobohedleolide is a diterpenoid compound. In rat models of paw edema and cotton pellet-induced granuloma, lobohedleolide (10 mg/kg) exhibited significant anti-inflammatory activity, with potency comparable to that of indomethacin [49]. Lysionotin is a flavonoid compound that exhibits significant bactericidal activity against Bacillus subtilis, Staphylococcus aureus, and methicillin-resistant Staphylococcus aureus [50]. Based on these findings, it can be inferred that the elevated concentrations of kaempferol 3,7,4'-trimethyl ether, kumatakenin, lobohedleolide, and lysionotin observed in this study may contribute to enhancing antioxidant capacity, alleviating intestinal inflammation, and preserving intestinal barrier function.

In this study, differential metabolites between the CON and PM40 groups were significantly enriched in the steroid hormone biosynthesis. Steroid hormones play crucial roles in maintaining vital activities, regulating growth and development, modulating reproductive functions, and mediating immune responses [51]. Cui et al. [52] reported that in calves with BCoV-induced diarrhea, the steroid hormone biosynthesis was significantly enriched in feces, accompanied by a marked downregulation of metabolites such as corticosterone, cortisol, and pregnenolone sulfate. Similarly, Tang et al. [53] observed in a rat model of spleen deficiency-type IBS-D that most significantly altered fecal and serum metabolites exhibited downward trends; after intervention with fecal microbiota transplantation or Shenling Baizhu San, the levels of some metabolites partially recovered, and the steroid hormone biosynthesis remained significantly enriched in both feces and serum. These findings collectively suggest that the steroid hormone synthesis may serve as a key regulatory pathway in the onset and progression of diarrhea. In the present study, the significant upregulation of 2-methoxyestrone 3-glucuronide, estrone glucuronide, and testosterone glucuronide indicates that PM40 treatment may modulate the conjugation, transport, and metabolic turnover of steroid hormone-related metabolites in calves. Correlation analysis of the selected absorbed bioactive components and the selected differential metabolites revealed that acacetin, chrysin, tectochrysin, dihydroartemisinic acid, and lysionotin are closely associated with metabolic remodeling in calves. However, it is currently not possible to establish a causal regulatory relationship between specific absorbed bioactive components and the differential metabolites. Further validation is required through fecal microbiota transplantation.

Gastrointestinal microbiota plays a fundamental role in regulating host physiology and maintaining health [54, 55]. Substantial evidence suggests that higher microbial diversity and abundance enhance the ability of hosts to resist pathogen colonization [56]. In contrast, microbial diversity and abundance typically decline under stress conditions [57]. In this trial, Chao and ACE indices for both ruminal and fecal microbiota in the PM40 group are higher than in the CON group, indicating enhanced microbial community richness, which aligns with previous findings [58, 59]. *Mediterranea massiliensis*, *Prevotella denticola*, *Duncaniella freteri*, and *Clostridium nexile* relative abundances were significantly increased in the PM40 group than in the CON group. Previous studies have demonstrated that *Mediterranea massiliensis* possesses polysaccharide-degrading capacity, expresses cellulosome structures, and produces acetate via the fumarate respiration pathway, enhancing energy harvest [60]. *Duncaniella freteri* is a fiber-degrading bacterium potentially involved in glycolysis and short-chain fatty acid biosynthesis [61]. *Clostridium nexile* can ferment dietary fiber to generate acetate [62], and its increased relative abundance in calves was closely linked to improved health status [63]. It is noteworthy that PM40 treatment significantly reduced the abundance of bacteria such as *Blautia producta* and *Alistipes putredinis*. Although some studies have suggested potential beneficial functions of these microorganisms under specific conditions [64, 65], their ecological roles are highly dependent on the physiological and pathological state of the host. Within the specific context of calf diarrhea, the proliferation of these bacterial taxa may be closely associated with pathological processes [66]. Correlation analysis between the selected differential metabolites and microbiota revealed that *Alistipes putredinis* and *Vescimonas fastidiosa* may be the primary bacterial responders to PM40 supplementation. However, these associations do not constitute direct evidence and warrant further validation through fecal microbiota transplantation to test this hypothesis.

In summary, PM supplementation alleviated diarrhea in calves by modulating the microbial community structure in the rumen and feces and by regulating serum steroid hormone synthesis. However, certain limitations of this study should be acknowledged. First, due to limitations in the experimental design, metabolomic and 16S rRNA sequencing analyses were performed only at the endpoint of the study. Consequently, the temporal dynamics of microbial community remodeling and metabolic profile changes during the onset and recovery of diarrhea could not be captured. Second, the absence of a positive control group prevents benchmarking the therapeutic efficacy of PM. Therefore, while the PM40 group significantly reduced the incidence of calf diarrhea under the present experimental conditions, its actual efficacy relative to existing interventions remains unclear. Future studies incorporating a positive control group are warranted to more comprehensively evaluate the feasibility of PM as an alternative strategy in calf production. Finally, the network pharmacology findings in this study were only preliminarily validated through molecular docking, and direct experimental evidence remains lacking. Further functional validation experiments targeting key molecules within the PI3K/AKT pathway in bovine intestinal tissue are warranted.

## Conclusion

This study showed that dietary PM supplementation (40 g/d) significantly reduced diarrhea incidence and improved jejunal villus morphology in preweaning calves. Integrated serum metabolome, rumen, and fecal microbiota analyses suggest that these beneficial effects may be linked to remodeling steroid hormone biosynthesis and the optimization of rumen and fecal microbial communities. Network pharmacology revealed that acacetin, chrysin, tectochrysin, dihydroartemisinic acid, and lysionotin may be potential active constituents. These findings provide a theoretical foundation for the future development of herbal mixtures as potential alternatives to conventional interventions in calf feeding, although the proposed mechanisms and key active components require further experimental validation.

## Supplementary Information

Additional file 1: Fig. S1. The frequency of diarrhea in Holstein calves as influenced by dietary supplements without polyherbal mixtures (PM) or with varying doses of PM during the experimental period.

## Data Availability

The datasets used and analyzed during the current study are available from the corresponding author on reasonable request.

## Abbreviations

BC: Betweenness centrality
CP: Crude protein
DC: Degree centrality
EC: Eigenvector centrality
LAC: Local average centrality
LEfSe: Linear discriminant analysis effect size
MCH: Mean corpuscular hemoglobin
MCHC: Mean corpuscular hemoglobin concentration
MCV: Mean corpuscular volume
MPV: Mean platelet volume
NC: Network centrality
PCoA: Principal coordinate analysis
PCT: Plateletcrit
PDB: Protein data bank
PDW: Platelet distribution width
P-LCC: Platelet large cell count
P-LCR: Platelet large cell ratio
PLT: Platelet
PM: Polyherbal mixture
PPI: Protein-protein interaction
TCVM: Traditional Chinese Veterinary Medicine
TS: Total solid
