# Supplementary material for: Mechanism of a polyherbal mixture alleviates calf diarrhea: an integrated network pharmacology, metabolomics, and microbiome study
Source: J Anim Sci Biotechnol. 2026 May 31;17:105. doi: 10.1186/s40104-026-01425-8 (PMC13222500; doi:10.1186/s40104-026-01425-8)
Supplement: Supplementary file 1 — Additional file 1: Fig. S1. The frequency of diarrhea in Holstein calves as influenced by dietary supplements without polyherbal mixtures (PM) or with varying doses of PM during the experimental period. [file 40104_2026_1425_MOESM1_ESM.docx]

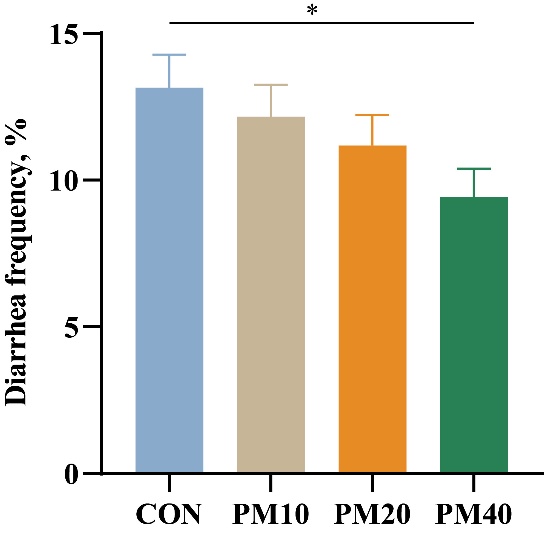


**Fig. S1** The frequency of diarrhea in Holstein calves as influenced by dietary supplements without polyherbal mixtures (PM; CON) or with varying doses of PM (PM10 = 10 g/d, PM20 = 20 g/d, PM40 = 40 g/d) during the experimental period (d 4–60) (*n* = 16 per treatment); ^*^*P* < 0.05
